# Does high-definition transcranial direct current stimulation change brain electrical activity in professional female basketball players during free-throw shooting?

**DOI:** 10.3389/fnrgo.2022.932542

**Published:** 2022-09-14

**Authors:** Luciane Aparecida Moscaleski, André Fonseca, Rodrigo Brito, Edgard Morya, Ryland Morgans, Alexandre Moreira, Alexandre Hideki Okano

**Affiliations:** ^1^Center of Mathematics, Computation, and Cognition, Federal University of ABC (UFABC), São Bernardo do Campo, Brazil; ^2^Neuroscience Applied Laboratory, Federal University of Pernambuco, Recife, Brazil; ^3^Edmond and Lily Safra International Institute of Neuroscience, Santos Dumont Institute, Macaíba, RN, Brazil; ^4^Department of Sports Medicine and Medical Rehabilitation, Sechenov First State Medical University, Moscow, Russia; ^5^Department of Sport, School of Physical Education and Sport, University of São Paulo, São Paulo, Brazil

**Keywords:** non-invasive brain stimulation, spectral analysis, neural efficiency, hypofrontality, neural proficiency

## Abstract

Differentiated brain activation in high-performance athletes supports neuronal mechanisms relevant to sports performance. Preparation for the motor action involves cortical and sub-cortical regions that can be non-invasively modulated by electrical current stimulation. This study aimed to investigate the effect of high-definition transcranial direct current stimulation (HD-tDCS) on electrical brain activity in professional female basketball players during free-throw shooting. Successful free-throw shooting (*n* = 2,361) from seven professional female basketball players was analyzed during two experimental conditions (HD-tDCS cathodic and sham) separated by 72 h. Three spectral bio-markers, Power Ratio Index (PRI), Delta Alpha Ratio (DAR), and Theta Beta Ratio (TBR) were measured (electroencephalography [EEG] Brain Products). Multi-channel HD-tDCS was applied for 20 min, considering current location and intensity for cathodic stimulation: FCC1h, AFF5h, AFF1h (−0.5 mA each), and FCC5h (ground). The within EEG analyses (pre and post HD-tDCS) of frontal channels (Fp1, Fp2, F3, F4, FC1, FC3) for 1 second epoch pre-shooting, showed increases in PRI (*p* < 0.001) and DAR (*p* < 0.001) for HD-tDCS cathodic condition, and in TBR for both conditions (cathodic, *p* = 0.01; sham, *p* = 0.002). Sub-group analysis divided the sample into less (*n* = 3; LSG) and more (*n* = 4; MSG) stable free-throw-shooting performers and revealed that increases in pre to post HD-tDCS in PRI only occurred for the LSG. These results suggest that the effect of HD-tDCS may induce changes in slow frontal frequency brain activities and that this alteration seems to be greater for players demonstrating a less stable free-throw shooting performance.

## Introduction

There are several methods to evaluate improvements in athletes' performance, particularly, a continuous search for ergogenic aids to boost sports performance (Banissy and Muggleton, 2013; Schubert and Astorino, 2013). Recently, the focus on searching for useful and effective ergogenic strategies has changed to the brain and to a better understanding of how it could limit/improve sports performance (Machado et al., 2019). In this sense, examining electrical brain activity using electroencephalography (EEG) is an approach aimed at improving performance understanding as well as athlete performance enhancement (Thompson et al., 2008). It has been hypothesized that identifying a distinctive EEG profile associated with a skilled performance may aid the relevant understanding of the cortical processes underlying top-level performance (Thompson et al., 2008).

EEG is a neuro-imaging method that presents excellent time-resolution which is required to accurately measure rapid changes in brain signal. Monitoring EEG in the field of sports science has recently emerged as a research area that may provide new insights into the nature of athlete performance (Comani et al., 2021) and the main mechanisms controlling and regulating the elite performance of high-level athletes. The quantitative EEG (qEEG) is considered a reliable instrument for detecting neural efficiency, transient hypofrontality, and neural proficiency that are proposed to explain optimal sports performance experiences (Holmes and Wright, 2017; Vickers and Williams, 2017; Filho et al., 2021).

Recently, Filho et al. (2021) conducted a meta-analytic review on changes in brain rhythm in optimal performance of self-paced sports (i.e., archery, Basketball free-throw, Golf putting, Pistol and Air-pistol shooting). The results of their study highlighted the role of alpha and theta activity in optimal performance, noting the theoretical concepts of neural efficiency, transient hypofrontality, and neural proficiency, and suggested that these are complementary neural mechanisms which may explain optimal performance. Moreover, in accordance with the transient hypo-frontality hypothesis (Dietrich, 2003, 2004), it has been previously suggested that neural activity is reduced in experts, which may be apparent in an elite athletes' brain that is characterized by more efficient resource distribution, more economic activity, and hypo-activation (Duru and Assem, 2018). According to this hypothesis, decreased frontal lobe functional activity may contribute toward explaining optimal performance experiences.

Thus, examining the EEG power frequency spectrum would advance knowledge of the neural correlates that underpin performance while also providing important information for applied neuro-feedback interventions (Pacheco, 2016; Xiang et al., 2018) and neuromodulatory strategies aimed at increasing the probability of optimal performance experiences (Morya et al., 2019; Moreira et al., 2021a). Therefore, adopting the qEEG index ratio between slower and faster frequencies such as the Delta Alpha Ratio (DAR), the Power Ratio Index (PRI; delta + theta/alpha + beta), and the Theta/Beta Ratio (TBR) (Brito et al., 2021) would advance this knowledge in elite athletes.

Considering the theoretical notions of neural efficiency, transient hypo-frontality, and neural proficiency, in particular during self-paced sports with closed and sports-specific skills, such as performing a free-throw in basketball, it would be reasonable to hypothesize that perturbation in the excitability of the cortex would occur. In particular, the brain areas involved with cognitive control (top-down control), named the prefrontal cortex (PFC), may alter the spontaneous neuronal activity, which may also affect free-throw performance in professional basketball players. Furthermore, it has been suggested that the dorsolateral prefrontal cortex (DLPFC) is involved in the inhibition responses (Miyake et al., 2000), and previous brain stimulation and neuroimaging studies have shown that cognitive control functions and executive control are primarily processed in the left DLPFC (Smith et al., 1998; MacDonald et al., 2000; Fregni et al., 2005). Thus, it may be hypothesized that modulating the excitability of the left DLPFC in basketball players may alter cognitive control during a free-throw shooting task. Nevertheless, there are no data examining the activity of the PFC or DLPFC during free-throws in professional female basketball players (Klostermann et al., 2018), and the effects of neuro-modulatory interventions over the left DLPFC on brain activity associated with free-throw shooting in professional basketball players are also unknown.

Transcranial direct current stimulation (tDCS) is non-invasive brain stimulation that involves a neuro-modulatory application of a weak electric current (up to 4 mA) using electrodes attached to the scalp over the brain region of interest (Bikson et al., 2016). Traditionally, the conventional application of the tDCS, placing the anode electrode near the nominal target (anodal tDCS, a-tDCS) is thought to increase neuronal excitability and plasticity, whereas placing the cathode electrode near the nominal target (cathodal tDCS, c-tDCS) is understood to induce the opposite effect (Nitsche and Paulus, 2001). Due to a non-linear dose-response (e.g., anodal inhibiting or cathodal exciting) (Batsikadze et al., 2013; Jamil et al., 2017; Esmaeilpour et al., 2018) and the interaction of active and reference electrodes (Bikson et al., 2010) it may be assumed that this polarity-dependent rationale is nevertheless over-simplistic. Indeed, for this “conventional” tDCS application, in which the electrical current is applied through large rectangular electrode pads of conductive material, it has been demonstrated that the current path is diffuse, and the peak electrical field is not strictly under the electrode, therefore generating low focality (Bikson et al., 2010, 2012; Moliadze et al., 2010). To improve the special focality, using a ring electrode rather than the conventional rectangular pad, the “high-definition” tDCS (HD-tDCS) was developed and proposed (Datta et al., 2008, 2009). Previous studies have shown that HD-tDCS, compared to conventional tDCS, present greater focality, stimulating gyri-level precision (Edwards et al., 2013; Villamar et al., 2013).

Despite knowledge advancement into the effects of conventional tDCS on performance (i.e., Okano et al., 2015; Machado et al., 2019; Valenzuela et al., 2019) and recovery processes (Moreira et al., 2021a,b) in athletes, and the comparative effects of conventional versus HD-tDCS in athletes (Machado et al., 2021), it is still unknown whether the use of HD-tDCS would affect brain electrical activity during free-throw shooting in female professional basketball players. Specifically, it may be hypothesized that cathodal stimulation over the left DLPFC would suppress executive control, including working memory activity, leading to a shift in dominance from the explicit memory system to the implicit system (McKinley, 2014). Consequently, promoting performance that may be more automatic, stable, and efficient. Therefore, the main aim of this study was to investigate the effect of HD-tDCS on brain electrical activity in professional female basketball players during the preparation for free-throw shooting. It was hypothesized that HD-tDCS may alter brain oscillation during the preparation for free-throw shooting in professional female basketball players.

## Materials and methods

### Participants

Nine elite professional female basketball players from the same team (33.5 ± 5.7 years old) participated in this study. The analyzed players were from a team ranked 1^st^ in the National Female Basketball league during the study period. The selected sample was classified as “elite athletes,” due to >10 h per week of training volume, having professional athlete status, and participating in National and International competitions (McKinney et al., 2019). The study was conducted during the beginning of the competitive season (season 2020; before the occurrence of the COVID-19 quarantine). The inclusion criteria were as follows: The participants were members of the assessed basketball team, they had regular participation in the team training program, and no disease or injury was reported during the study period that may interfere with performing the protocol and signing the informed consent form. The exclusion criteria were: disinterest during the test protocol, missing data points, taking any medication that could affect the central nervous system, contra-indication for tDCS (i.e., no implanted metal in the head, pacemaker, seizures, lesions on the scalp or head). The players received thorough instructions on the experimental design and signed the consent and image rights forms. Data from two players were excluded from the final analyses due to errors in EEG acquisition. Approval for the study from the club was obtained and ethics was granted by the Ethics Committee of Federal University of ABC (CAAE: 08070819. 1.0000.5594). All procedures involving human participants were conducted in accordance with the 1964 Helsinki declaration.

### Experimental design

**Figure 1:**
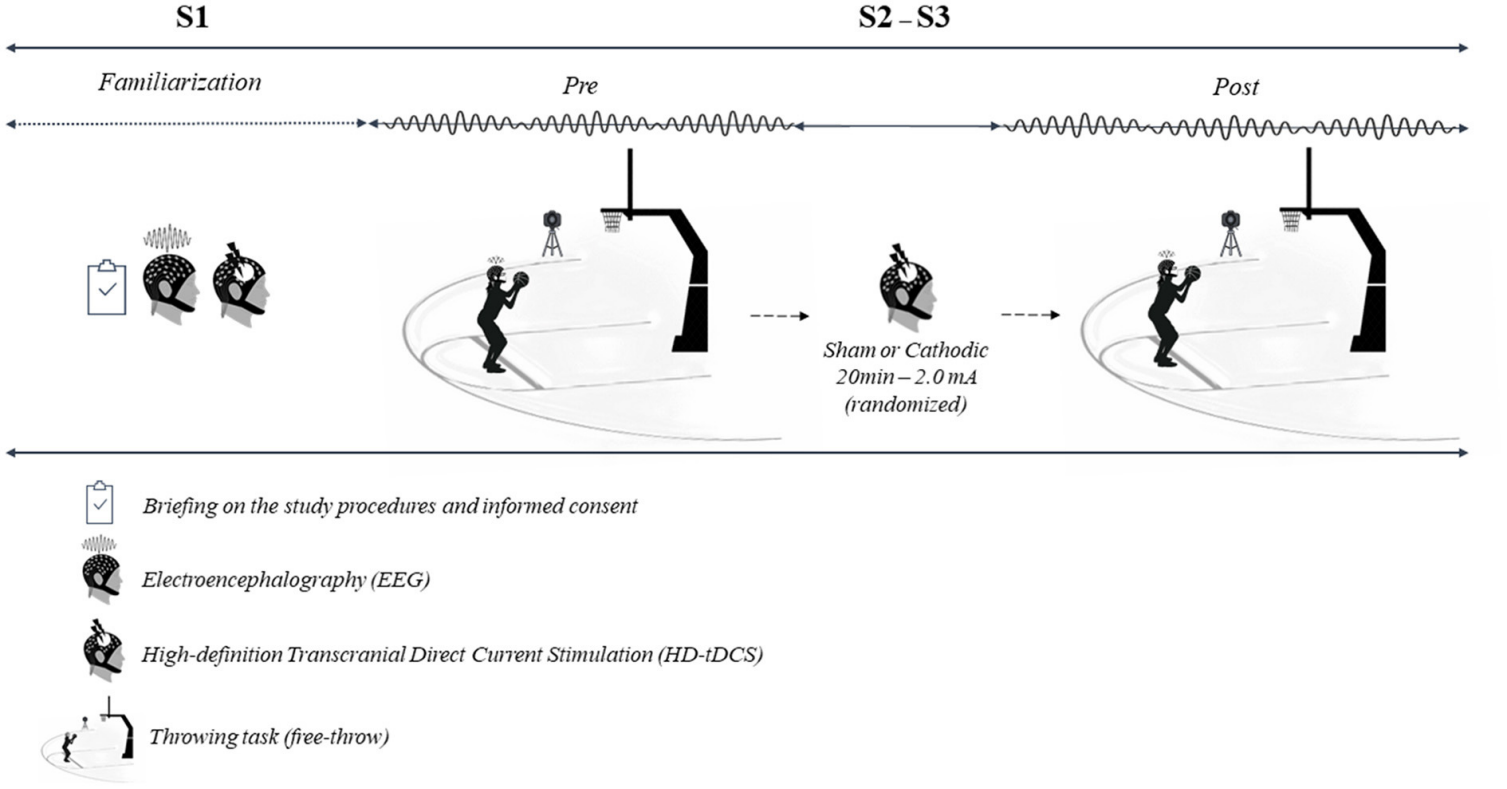
Study design.

This research was a randomized, crossover, and double-blind experimental design (Figure 1). Three test sessions were carried out. The first session was to familiarize all participants with the test protocol. The players were informed of the study protocol, had any questions answered regarding characterization, familiarized themselves with the EEG and HD-tDCS, and then signed the informed consent form. In the subsequent two sessions, participants performed the experimental shooting protocol. The players performed 200 free-throw shots in each session (the shot distance was 4.23 m; i.e., the free-throw line according to official FIBA rules) from in front of the basket. Players performed 100 free-throws (pre- HD-tDCS) and in a randomized order, received either cathodal HD-tDCS or active sham tDCS for 20 min and then performed another 100 free-throws (post-HD-tDCS). All players were placed in the two conditions (cathodic and sham HD-tDCS). A posteriori, the sample was sub-divided into less-stable (LSG) and more-stable (MSG) groups considering the changes in successful shots from pre- to post-sham. The within-subjects standard deviation was used as an index of measurement error (Bland and Altman, 1996). The group median for the within-subjects standard deviation (1.41) was adopted to divide (median split) players into the sub-groups. Therefore, the LSG was composed of players who presented differences in more than two successful shots from pre- to post-sham.

### Video analytics and free-throw shooting performance

The shooting protocol was recorded to quantify the free-throw shooting performance (successful shots), using a camera with 60 Hz of frequency acquisition (60 ms per video frame) positioned on the side-line of the basketball court.

### Electroencephalography, data processing, and analysis

Cortical electrical activity was measured using 64 active electrodes on the scalp's surface, connected to a Brain Products 24-bit BrainVision actiChamp amplifier, with an acquisition frequency of 1,000 Hz during the preparation for the free-throw shooting and throughout the task. However, as the main aim of the current study was to investigate the effect of HD-tDCS on brain electrical activity in professional female basketball players during the preparation for free-throw shooting, the spectral bio-markers analyses were conducted for 1-s epoch pre-shooting. This 1-s time window was determined based on the results of a frame-by-frame video analysis of each player's free-throw shooting. Three expert basketball trainers observed that the time duration from the beginning of the shooting preparation to the last frame the ball left the hand was no longer than 1 s. This finding is supported by Aglioti et al. (2008) who used movies of free shooting performed by professional basketball players with preparation and shooting with <1 s. The preparation for free-throw shooting in the present study was defined therefore as the player's complete movement until the instant the ball left her hand. Standard placement of the electrodes for EEG recording followed the international 10/20 EEG system. The EEGLab toolbox performed signal processing in MATLAB^®^ R2020b for Windows. In the sequence of pre-processing the EEG signals, the sampling frequency was reduced to 500 Hz, and high-pass and low-pass filters of 0.5 and 50 Hz were applied, respectively. Independent component analysis (ICA) was used to remove artifacts in continuous data. The absolute spectral power was calculated using the Welch estimate for frequency bands: delta δ (0.5–≤4 Hz), theta θ (>4–≤8 Hz), alpha α (>8–≤13 Hz), beta β (>13–≤30 Hz) and gamma γ (>30 Hz), where the values of the relative spectral powers and the spectral bio-markers (PRI, DAR, and TBR) were calculated for 1-s epoch pre-shooting (the preparation for the free-throw shooting), considering, PRI = delta+theta/alpha+beta, DAR = delta/alpha and TBR = theta/beta, respectively. For all analyses, successful free-throws were considered.

### High-definition transcranial direct current stimulation

**Figure 2:**
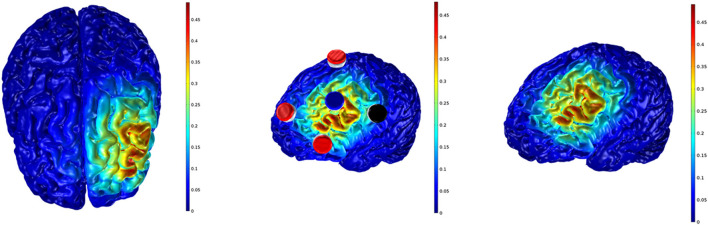
Electrodes setup and voltage field simulation. Cathodal stimulation; gray matter electric field: 0.48 V/m.

High-definition transcranial direct current stimulation (Soterix Medical, New York, NY) was employed to produce cathodal and sham stimulation over the DLPFC for 20 min with a ramp up and down of 30 s. Participants were not aware of the tDCS condition received. The HD-tDCS electrodes were fixed into an EEG cap with 64 channel positions (Acticap; Brain Products, Munich, Germany) designed for simultaneous EEG-tDCS measurements. Each plastic casing was filled with approximately 2 ml of HD-tDCS gel (Soterix Medical, New York, NY) to connect the electrode and scalp. The HD-tDCS only commenced when the impedance was < 10 kOhms. Five ring Ag-AgCl electrodes connected to a tDCS device (MxN, Soterix Medical, New York, NY) were used for all tDCS conditions. Transcranial direct current stimulation montage was determined based on computational modeling using a finite element model of current brain flow (Figure 2). The cathodic stimulation were: FCC1h, AFF5h, AFF1h (−0.5 mA each) and FCC5h (ground) for 20 min. For sham stimulation (placebo effect), the current was applied at the beginning (30 s) and end (30 s) of the stimulation period (ramp up and down of 30 s). The stimulation electrodes were maintained in the same position used in the cathodic condition. It was expected that the subjects would experience the same sensations related to cathodic ramping up and down. This procedure is an auto-sham, which is automatically calculated and produces a sham waveform based on the indicated “real” waveform. For example, for a corresponding real waveform of 2.0 mA and 20 min, auto-sham will provide a ramp up and down to 2.0 mA at the start of stimulation, and again after 20 min, with the timer automatically adjusted such that the total run time is exactly matched to the real case, the cathodic stimulation condition in the case of the present study. There was an interval of 72 h between sessions. At the conclusion of each session, participants completed a questionnaire indicating the sensations and intensity felt during the stimulation proposed by Fertonani et al. (2015).

### Statistical analysis

Data are reported as mean and standard deviation (SD) or as median and interquartile range. To attenuate the well-known inherent variability observed in electroencephalography data, for each subject value-distribution we considered the middle 50% (between the 25 and 75% percentiles). Therefore, only the data from the spectral bio-markers analysis (PRI, DAR, and TBR) during the preparation for free-throw shooting (1-s epoch pre-shooting) that were between 25 and 75% percentiles were retained for analysis. A non-parametric Friedman test was used for within-condition analysis (pre- and post-tDCS conditions, for PRI, DAR, and TBR). Following sample analysis, sub-group analyses were conducted. The sample was divided LSG and MSG shooting performers. Another non-parametric Friedman test was used for within-group analysis (pre- and post- each tDCS condition, for PRI, DAR, and TBR). For both analyses (sample and sub-group analysis), the level of significance was set at 1% (*p* ≤ 0.01). Moreover, Effect sizes (ESs) were calculated as the standardized mean difference to determine the meaningfulness of the difference between within (within-sub-group analysis) changes in successful free-throw shooting from pre – to post- HD-tDCS conditions (sham and cathodic), corrected for bias using Hedge's formula (Hedge's g uses pooled weighted standard deviations), and presented with 95% Confidence Limits (CL) (Batterham and Hopkins, 2006). ESs with values of 0.2, 0.5, and 0.8 were considered small, medium, and large differences, respectively (Cohen, 1988). Data were analyzed using Microsoft Excel (Microsoft™, USA).

## Results

### Free-throw performance

**Figure 3:**
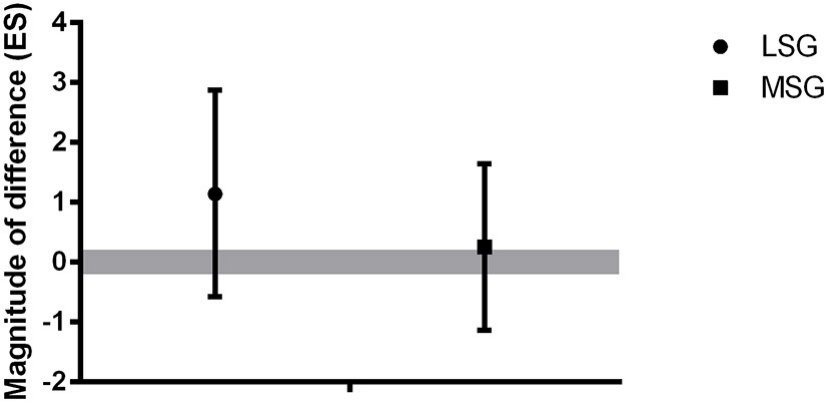
The magnitude of differences between changes in free-throw shooting from pre – to post-HD-tDCS sham and cathodic for LSG and MSG. LSG, Less stable group; MSG, More stable group. Positive ES, magnitude of increases in successful free-throw shooting from pre- to post-cathodic. Negative ES, magnitude of decreases in successful free-throw shooting from pre – to post-cathodic. Gray bar denotes an effect size (ES) > 0.20.

Table 1 presents the individual successful free-throw shooting for sham and cathodic conditions highlighting the players included in LSG and MSG. Figure 3 shows the magnitude of differences (ES) between changes in successful free-throw shooting from pre – to post-HD-tDCS sham and cathodic for LSG and MSG. The data for LSG revealed a large difference (ES = 1.25) in changes in successful shooting from pre – to post-cathodic condition compared to sham. However, the ES for MSG showed small changes (ES = 0.25) in successful shooting after the cathodic intervention compared to sham.

**Table 1:** Individual successful free-throw shooting for sham and cathodic HD-tDCS conditions.

	**Sham**		**Cathodic**	
	**Pre**	**Post**	**Pre**	**Post**
Player 1 (LSG)	85	92	89	92
Player 2 (LSG)	86	81	78	88
Player 3 (LSG)	78	74	70	74
Player 4 (MSG)	91	91	88	88
Player 5 (MSG)	91	91	97	96
Player 6 (MSG)	83	84	81	86
Player 7 (MSG)	78	80	74	75
The whole group mean ± SD	84.57 ± 5.38	84.71 ± 6.87	82.43 ± 9.43	85.57 ± 8.24
Group mean ± SD (LSG)	83.00 ± 4.36	82.33 ± 9.07	79.00 ± 9.54	84.67 ± 9.45
Group mean ± SD (MSG)	85.75 ± 6.40	86.50 ± 5.45	85.00 ± 9.83	86.25 ± 8.66

LSG, Less stable shooting performers; MSG, More stable shooting performers; SD, Standard Deviation.

### Power ratio index, delta to alpha ratio, and theta to beta ratio

**Figure 4:**
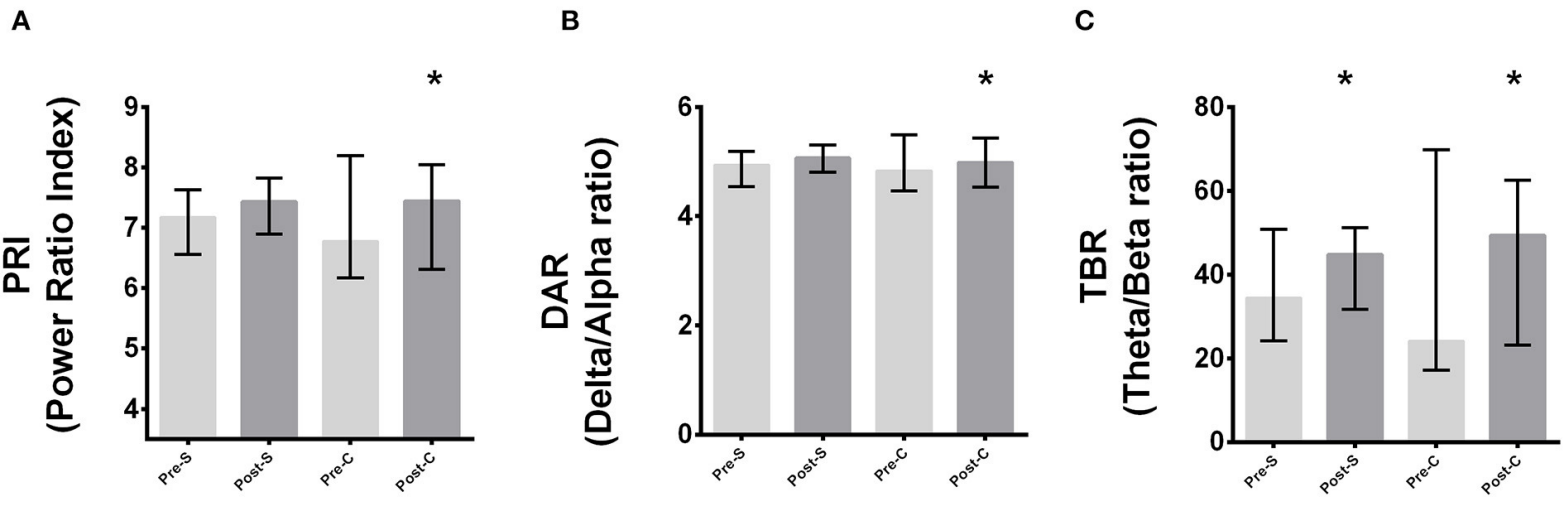
Intra-condition analyses of PRI, DAR, and TBR considering channels Fp1, Fp2, F3, F4, FC1, FC3. *Significant difference from pre - to post-stimulation for **(A)** PRI, **(B)** DAR, **(C)** TBR (*p* < 0.001). Pre-S, Pre-Sham; Post-S, Post-Sham; Pre-C, Pre-Cathodic; Post-C, Post-Cathodic. PRI, Power Ratio Index; DAR, Delta to Alpha Ratio; TBR, Theta to Beta Ratio.

To verify the effect of HD-tDCS (pre- and post-HD-tDCS), specifically in the frontal area, a set of 6 channels (Fp1, Fp2, F3, F4, FC1, FC3) was used. During the preparation for shooting, PRI (*p* < 0.001) and DAR (*p* < 0.001) spectral bio-markers increased in tDCS cathodic condition. Indeed, TBR significantly increased from pre- to post-interventions for both conditions (cathodic, *p* = 0.01; sham, *p* = 0.002). The within (intra-condition) analyses are shown in Figure 4, for PRI (A), DAR (B), and TBR (C) bio-markers. Data are reported as median and inter-quartile ranges (25–75%).

**Figure 5:**
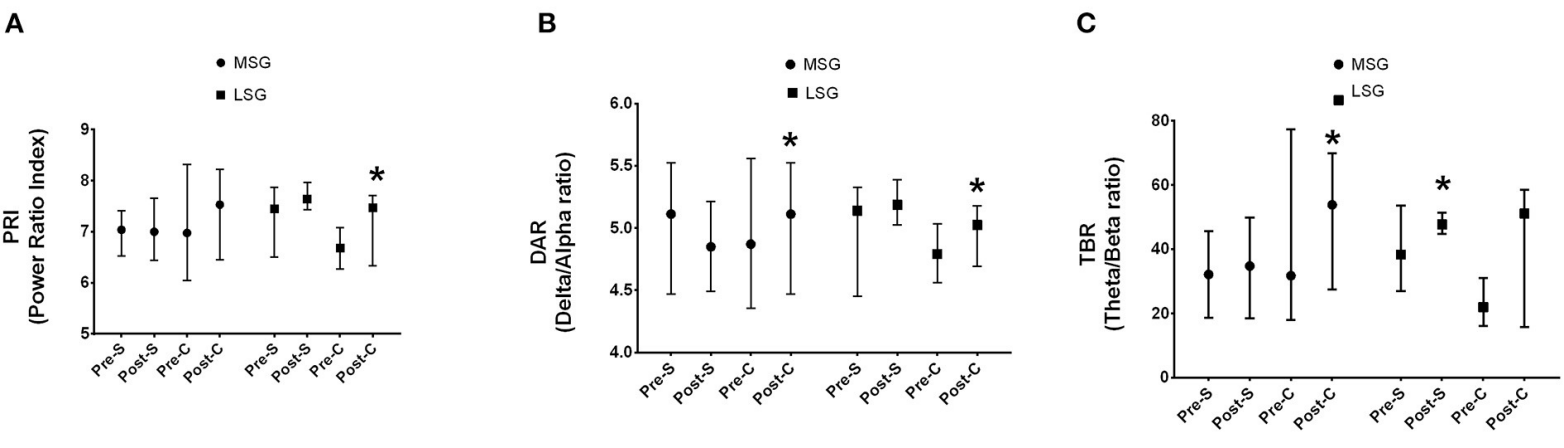
Sub-group analyses considering Fp1, Fp2, F3, F4, FC1, FC3 channels. **(A)** PRI, **(B)** DAR, **(C)** TBR. *Significant difference between pre - to post-stimulation (*p* < 0. 001). LSG, Less stable group; MSG, More stable group. Pre-S, Pre-Sham; Post-S, Post-Sham; Pre-C, Pre-Cathodic; Post-C, Post-Cathodic. PRI, Power Ratio Index; DAR, Delta to Alpha Ratio; TBR, Theta to Beta Ratio.

Figures 5A–C present results for the sub-group analyses for PRI, DAR, and TBR bio-markers, respectively (frontal area; 6 channels {Fp1, Fp2, F3, F4, FC1, FC3}). PRI (*p* < 0.001) spectral bio-marker significantly increased in tDCS cathodic condition only for the LSG. DAR increased significantly for LSG (*p* < 0.001) and MSG (*p* = 0.01). Indeed, TBR significantly increased from pre- to post-interventions in tDCS cathodic condition for the MSG (*p* = 0.03) and in sham condition for LSG (*p* < 0.001). Data are reported as median and inter-quartile ranges (25–75%).

## Discussion

This study aimed to investigate the effect of HD-tDCS on brain electrical activity in professional female basketball players during free-throw shooting. The main findings of this study were that HD-tDCS induced changes in slow frontal frequency brain activities. Specifically, the cathodic, but not the sham condition, was effective to increase PRI and DAR spectral bio-markers considering the sample group analysis, indicating changes (increases) in the slow frontal frequency brain activities. Indeed, interestingly, for TBR, the increases were seen in both conditions (cathodic and sham). Additionally, the sub-group analysis, which divided the group into less and more stable performers in shooting, showed that the effect of cathodic HD-tDCS occurred in both groups for DAR, but only in LSG for PRI. For TBR, however, there was an effect of sham condition in LSG and of cathodic condition in MSG.

To our knowledge, this is the first study to investigate the effect of cathodal tDCS over the left DLPFC on brain electrical activity in professional female basketball players during free-throw shooting. Our hypothesis regarding the effect of the cathodal stimulation over the left DLPFC was confirmed. The increases in PRI, DAR, and TBR from pre- to post- tDCS suggest that professional female basketball players were performing the free-throw shooting task through a more automatic pathway reducing the activity of the explicit memory. Thus, this change may be associated with neural mechanisms reflecting a smarter working brain, free from (or better regulating) negative thoughts and doubts which might negatively impact the successful execution of the specific motor action.

In this sense, it should be highlighted that recent studies have reported an increased theta activity in the frontal lobe under-pinning optimal performance experiences in motor and cognitive tasks (di Fronso et al., 2016; Katahira et al., 2018) and proposed that this increase in theta activity is considered a marker of “brain busy-ness” (Pacheco, 2016). Furthermore, Bertollo et al. (2016) proposed an alternative framework for the neural efficiency hypothesis, which has been called the neural proficiency hypothesis. This framework proposed that athletes need to engage in efficient and effortful processing to consistently achieve high-level performance. This assumption implies that performing the task and combining automatic and deliberative thinking in a well-balanced way will achieve optimal performance.

As the quantitative EEG (qEEG) is regarded as a reliable instrument to detect neural efficiency, transient hypofrontality, or neural proficiency, and, as suggested by Filho et al. (2021), these theoretical notions are likely complementary neural mechanisms that maintain optimal performance, therefore, the results of the present study significantly add to the existing scientific literature. These results indicate the necessity for qEEG usage during sports tasks examining neural mechanisms and the theoretical notions associated with such explanations of these neural mechanisms or neural signatures involved in the preparation of a motor task, as examined herein. In addition, the authors also strongly suggest that the possibility of adopting HD-tDCS as a sports-specific intervention to modulate mental states with the aim of inducing a more automatic and efficient behavior is needed.

Moreover, the present results suggest that data from PRI and DAR bio-markers seems to be more reliable than TBR in order to investigate the effects of HD-tDCS on brain electrical activity using the qEEG index ratios. Changes in TBR from pre- to post-HD-tDCS were seen in both conditions (cathodic and sham), suggesting a possible occurrence of indirect neurobiological effects on this bio-marker. The likelihood of occurring neurobiological effects during the sham condition was proposed by Fonteneau et al. (2019). The authors referred to those effects which might be related to the application of weak electrical currents during the sham condition, as *direct neurobiological effects*. Indeed, Nikolin et al. (2018) examined the EEG activity of participants who were randomized across five groups to receive 15 min of bifrontal conventional tDCS at different current intensities, during and after a working memory task. They demonstrated that the P3 amplitude presented biological effects for all examined conditions, including sham (1 mA stimulation, 2 mA stimulation, and a sham condition [0.034 mA]). This effect might have occurred for the TBR index, despite the use of HD-tDCS, rather than the conventional tDCS as adopted in the study of Nikolin et al. (2018). Speculatively, it might be argued that the TBR is more sensitive to these neurobiological effects from sham and therefore, an index less reliable than PRI and DAR to be used to examine brain oscillation changes from HD-tDCS in female basketball players. This result opens an interesting venue for research examining the use of qEEG index ratios in athletes, and in particular, their dynamics changes for HD-tDCS application.

The findings of the present study suggest that the cathodical stimulation over the left DLPFC would lead to an increase in slow brain frequency activities (notably, for PRI and DAR), that in turn, has been suggested as a state where optimal performance may be attained. For example, Chuang et al. (2013) showed that a prevalence of slow waves in the frontal regions was observed at the beginning of the successful throw period in basketball. Moreover, the presence of low-frequency oscillations (delta and theta) has been associated with event-related desynchronization (ERD), as the amplitude decreases in alpha and beta waves during the preparation and execution of motor activities (Pfurtscheller and Aranibar, 1979). Indeed, this top-down regulation of behavior prior to motor execution has been shown to improve performance and is inferred to be an influential element for elite athletes in competitive environments (Chuang et al., 2013).

Additionally, the present results from the sub-group analysis highlighted the likelihood that having effective results from the non-invasive brain stimulation intervention for players who are less stable in shooting performance. This is a unique and interesting finding of the present study that should be emphasized. This result may suggest that even in a group of professional female basketball players, there is scope to improve in the free-throw shooting performance, as the data showed a large difference (ES = 1.25) in changes in successful shooting from pre – to post-cathodic condition compared to sham, in LSG. It might be hypothesized that those players who present more variability (less consistency) in free-throw shooting might gain meaningful benefits from the application of this intervention and the consequent changes in brain oscillation. However, future studies should investigate the association between changes in slow brain frequencies from HD-tDCS interventions with alterations in performance for both, closed-skill tasks and open-skill tasks with different complexity levels and constraints, using a large sample size and with different basketball players' levels to test this hypothesis.

Despite the relevance, uniqueness, and novelty of the current findings, some limitations should be considered when generalizing the results. In this study, the examined players were all from a single team, and due to the small sample size, this investigation may be considered a case study in top-professional female basketball players. Studies analyzing elite athletes often have an inherent reduced sample size, which in turn, may affect the power of the results and their generalizations. However, it is imperative to mention that the number of observation units (number of free-throw shots) were high and allowed a robust qEEG analysis to be conducted and a comparison of brain rhythms between pre- to post-HD-tDCS to occur. Indeed, considering the scant information available regarding possible professional player differences, further comparative studies assessing the effect of HD-tDCS on qEEG in female and male basketball players and athletes in general, are needed to elucidate this research area. Moreover, future studies with larger sample populations examining other tDCS montages in teams from different distinct levels would be beneficial. Furthermore, investigating possible variations in female basketball players from differing competition standards [i.e., regional players, state players, national and international players] should be conducted to add novel findings on this topic.

In summary, this study suggests that HD-tDCS may induce changes in slow frontal frequency brain activities in the preparation for free-throw shooting and that these alterations seem to be greater in players demonstrating higher variability in free-throw shooting performance. Moreover, the present results also suggest that employing HD-tDCS and EEG in combination during a closed-specific sports skill, such as free-throw shooting used in the present study, may potentially provide benefits in knowledge advancement in the neural mechanisms supporting elite-level athletic performance. Indeed, adopting the qEEG ratio index, for PRI and DAR, between slower and faster frequencies may contribute to further examination into the changes in brain rhythms related to optimal performance.

## Data availability statement

The original contributions presented in the study are included in the article/supplementary materials, further inquiries can be directed to the corresponding author.

## Ethics statement

The studies involving human participants were reviewed and approved by Ethics Committee of Federal University of ABC. The patients/participants provided their written informed consent to participate in this study.

## Author contributions

Conceptualization and research design: LM, EM, AM, and AHO. Data acquisition and/or analysis: LM, EM, AF, RB, AM, and AHO. Data interpretation: LM, EM, AF, RB, RM, AM, and AHO. Manuscript draft: with the collaboration of all authors. Substantial revision: all authors reviewed and approved the manuscript.

## Funding

AM is supported by CNPq (304269/2021-2). AHO is supported by CNPq (312489/2021-8). EM is supported by CNPq (315054/2021-2). AM, AHO, EM, and LM was supported by Brazilian Institute of Neuroscience and Neurotechnology (BRAINN/CEPID-FAPESP, Process: 2013/07559-3).

## Conflict of interest

The authors declare that the research was conducted in the absence of any commercial or financial relationships that could be construed as a potential conflict of interest.

## Publisher's note

All claims expressed in this article are solely those of the authors and do not necessarily represent those of their affiliated organizations, or those of the publisher, the editors and the reviewers. Any product that may be evaluated in this article, or claim that may be made by its manufacturer, is not guaranteed or endorsed by the publisher.
